# Comparison of hand-sewn versus mechanical esophagogastric anastomosis in esophageal cancer: Protocol for a systematic review and meta-analysis

**DOI:** 10.1097/MD.0000000000015676

**Published:** 2019-06-07

**Authors:** Yang Wang, Xiangwei Zhang, Yuanzhu Jiang, Guoyuan Ma, Zhaoyang Wang, Xianbiao Xue, Shaowei Sang, Lin Zhang

**Affiliations:** a Department of Medical Imaging; b Department of Thoracic Surgery, Shandong Provincial Hospital Affiliated to Shandong University, Jinan; c Department of Thoracic Surgery, Juye County People's Hospital, Juye; d Clinical Epidemiology Unit, Qilu Hospital of Shandong University, Jinan, People's Republic of China.

**Keywords:** anastomosis, esophageal cancer, hand-sewn, mechanical staples, meta-analysis, systematic review

## Abstract

**Background:**

Many studies have been conducted to compare the hand-sewn and mechanical staples in esophageal cancer (EC) patients who received esophagogastric anastomosis. However, the results remain controversial. Hence, the purpose of the meta-analysis is to evaluate the impact of different anastomosis methods on the early and long-term outcomes.

**Methods:**

We will perform a systematic electronic search of the PubMed, Embase, Cochrane Library, Web of Science for relevant articles published in English language. Pooled odds ratios will be calculated for the effect on discrete variables including anastomotic leakage, anastomotic strictures, 30-day mortality, quality of life, cardiac and pulmonary complications. The weighted mean difference was calculated for the effect size on continuous variables such as operative time and bleeding amount. We will use the software Review Manager 5.3 and STATA 14.0 to perform the meta-analysis to calculate the data synthesis.

**Results:**

The review will provide a high-quality synthesis of current evidence of the impact of different anastomosis methods on postoperative course in ECs after esophagectomy. The results will be published in a peer-reviewed journal.

**Conclusion:**

This systematic review and meta-analysis will compare the different anastomosis methods in EC patients. The results will better offer some specific suggestions for esophagogastric anastomosis.

**PROSPERO registration number:**

This systematic review protocol has been registered in the PROSPERO network (No. CRD 42019109523).

## 1 Introduction

Esophageal cancer (EC) is one of the most common gastrointestinal malignancies.^[1]^ The incidence of EC varies according to different geographic locations. Squamous cell carcinoma (SCC) and adenocarcinoma (AC) are the 2 dominant pathological types. SCC is prevalent in eastern countries while AC is more common in western countries.^[2]^ With the development of multiple disciplinary treatments, the prognosis of EC has been improved remarkably. However, the survival of EC patients is still poor even after apparent radical treatment with a 5-year survival rate of 34% to 47%.^[3]^ Although chemotherapy and radiotherapy are always used before and after surgery to improve the survival rates in patients with advanced locoregional disease, surgical resection of the esophagus with en-bloc lymphadenectomy remains the cornerstone of treatment and provides the only chance of cure for patients with localized EC.^[4]^

The procedures of EC surgery include dissection of the diseased esophageal portion, formation of the gastric conduit and creation of the esophagogastric anastomosis. Esophagogastric anastomosis can be performed by hand sewn or with mechanical anastomotic staples.^[5]^ Traditionally, the hand-sewn anastomosis can be fashioned using 1, 2, or even 3 layers of interrupted sutures.^[6]^ The mechanical anastomosis can be conducted by using circular or linear staples.^[7]^ The techniques of esophagogastric anastomosis for reconstruction are complex and associated with postoperative complications. Early postoperative complications such as anastomotic leakage could lead significant morbidity and mortality. Late postoperative complications including anastomotic strictures could cause negative impact on the patient's quality of life.

The rate of postoperative complications is still high though the anastomosis skills have been improved greatly and the method of anastomosis have been a great concern among surgeons. Recently, many randomized controlled trials (RCTs) have been conducted to compare the traditional hand-sewn anastomosis to the modern mechanical stapled anastomosis method.^[8,9]^ However, there is still no consensus. Here, we will perform a meta-analysis of RCT to compare the impact of anastomotic technique (hand sewn vs stapled) on patient outcomes after esophagectomy for EC.

## 2 Methods

### 2.1 Study registration

The Preferred Reporting Items for Systematic Reviews and Meta-Analyses Protocols (PRISMA-P) statement guidelines will be followed in the protocol.^[10]^ This systematic review and meta-analysis has been registered on PROSPERO with registration number: CRD 42019109523. Ethical approval is not required because this is a study based on aggregate data and did not involve humans.

#### 2.1.1 Data sources and search strategy

We will perform a systematic electronic search of the PubMed, Embase, Cochrane Library, Web of Science to identify articles for inclusion in our meta-analysis. Both full text and MeSH search for keywords were used. The main search terms include ’esophageal cancer’, ‘esophagectomy’, ‘anastomosis’, ’hand-sewn’ and 'stapled’. MeSH headings including ’anastomosis’ (MeSH), ’hand-sewn’ (MeSH), 'stapled’ (MeSH), and ’esophagectomy’ (MeSH) were used in combination with the Boolean operators AND or OR. The reference lists and related articles in each identified publication were also reviewed for potential studies.

Search strategy of PubMed was as follows:

(1) (esophagectomy) OR (anastomosis);
(2) (esophageal neoplasm) OR (EC) OR (esophageal carcinoma) OR (EC);
(3) (hand-sewn) OR (stapled); (4) Step 1 AND step 2 AND step3.

### 2.2 Inclusion and exclusion criteria

Articles were included if they met the following criteria:

(1) RCTs;
(2) patients who underwent esophagectomy and esophagogastric anastomosis for esophageal squamous cell cancer and/or AC;
(3) comparison of hand-sewn and mechanic anastomosis.

Articles were excluded if they meet the following criteria:

(1) non-randomized trials, abstracts, letters, editorials, reviews, or case reports;
(2) studies were not available in English;
(3) studies had overlapping or repeat data;
(4) studies concerned non-human or non-clinical research;
(5) patients dealing only with benign esophageal diseases.

### 2.3 Data extraction and quality assessment

#### 2.3.1 Selection of studies

**Figure 1:**
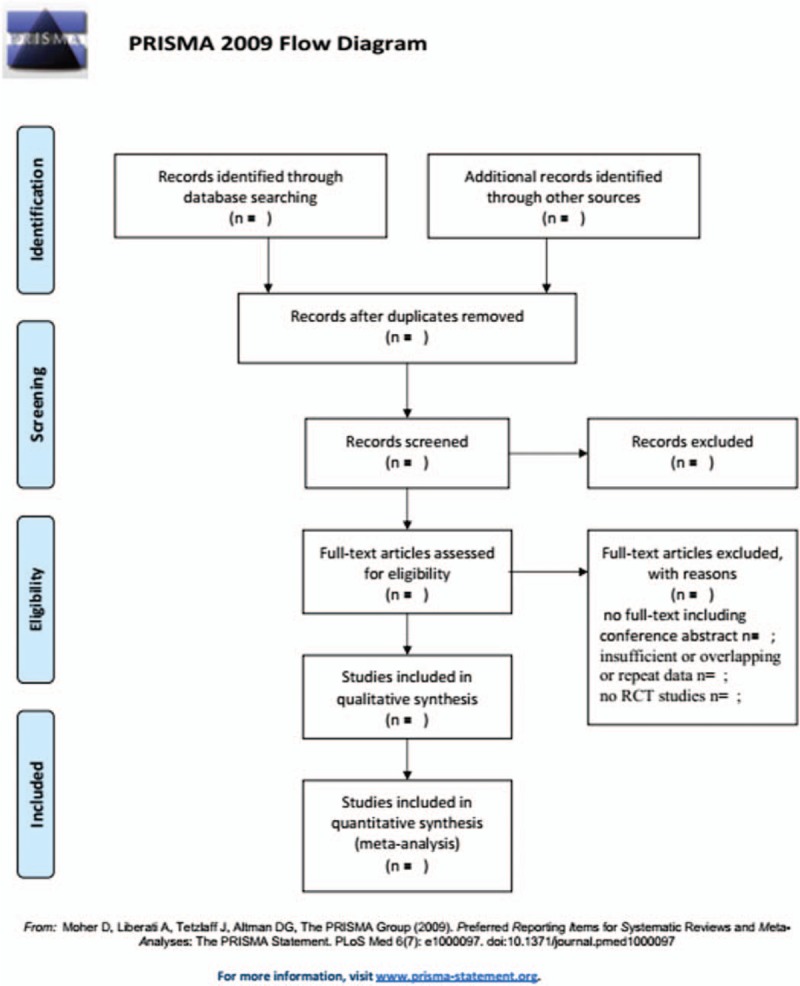
Flow chat of literature search and selection.

Two researchers (Yang Wang and Xiangwei Zhang) reviewed the eligible articles independently. If necessary, the third reviewer (Yuanzhu Jiang) will be consulted. Articles that could not be categorized based on title and abstract alone were retrieved for full-text review. The selection process will be summarized according to PRISMA flow diagram. (Fig. 1) They extracted data concerning details of patients’ characteristics, study methods, interventions, and outcomes by using the uniform data extraction forms. Trial data abstraction was also done independently and in duplicate.

#### 2.3.2 Data extraction and management

Two researchers (Yang Wang and Xiangwei Zhang) reviewed the eligible articles independently. Any disagreement will be solved by consensus or an arbiter (Yuanzhu Jiang). The odds ratios (ORs) and weighted mean difference were obtained directly from individual articles. Additionally, other clinic pathologic parameters were extracted using a unified form including author, year of publication, country of origin, total number of patients, age, sex, follow-up time, the anastomotic site, the hand-sewn method, the brand, and size of staples.

#### 2.3.3 Assessment of quality in included studies

The quality of each trial was assessed using the Jadad scoring system.^[11]^ Two reviewers (Yang Wang and Xiangwei Zhang) will independently assess the quality of each study and any conflicts disagreements in quality assessment were resolved by joint discussion.

#### 2.3.4 Measures of effects

Pooled ORs and 95% confidence intervals (CIs) were calculated for the effect on discrete variables including anastomotic leakage, anastomotic strictures, 30-day mortality, quality of life, cardiac and pulmonary complications. The weighted mean difference was calculated for the effect size on continuous variables such as operative time and bleeding amount.

#### 2.3.5 Management of missing data

We will contact the corresponding author to request any inadequate and missing data by E-mail if some data are missing in several included studies. If the data are still incomplete, we will perform data synthesis through available information and address the potential impact of missing data on the pooled results in the discussion parts.

#### 2.3.6 Assessment of heterogeneity

Assessment of heterogeneity between the included studies will be conducted to evaluate the feasibility of meta-analysis. The Cochran *Q* test and Higgins *I*^2^ method will be used to assess the heterogeneity.^[12]^ A *P* < .05 for *Q* test or *I*^2^ > 50% for *I*^2^ test suggested significant heterogeneity in the literature, while a *P* > .05 for *Q* test or *I*^2^ < 50% for *I*^2^ test indicated no heterogeneity. In cases of substantial heterogeneity, we will perform subgroup analysis to explore the potential causes.

#### 2.3.7 Data synthesis

All the statistical analyses will be performed using Review Manager 5.3 and STATA statistical software version 14.0. Cochran *Q* test and Higgins *I*^2^ statistic were undertaken to assess the heterogeneity of the included studies. A *P* < .10 for *Q* test or *I*^2^ > 50% for *I*^2^ test suggested significant heterogeneity in the literature and a random-effects model (DerSimonian–Laird method) was used.^[13]^ Otherwise, the fixed-effect model (Mantel–Haenszel method) was adopted.^[14]^ All *P*-values were two-sided. A *P* < .05 was considered statistical significant.

#### 2.3.8 Subgroup analysis

If the necessary data are available, subgroup analysis will be conducted to determine the possible factors that may influence the results:

(1) hand-sewn versus circular staplers;
(2) hand-sewn versus linear staplers;
(3) circular staplers versus linear staplers.

#### 2.3.9 Sensitivity analysis

Sensitivity analysis will be conducted by omitting each single study every time to see the influence of the individual dataset on the pooled ORs. The results will not be substantially changed when any study is excluded if the pooled ORs are robust.

#### 2.3.10 Publication bias

Begg funnel plot and Egger test linear regression test will be conducted to evaluate the publication bias of the included studies.^[15,16]^ We will perform the “trim and fill” test for further analysis if publication bias is detected (*P* < .05 is considered statistically significant).

## 3 Discussion

With the advances in surgical techniques and perioperative management, the rate of mortality and morbidity has decreased greatly after extensive esophagectomy surgery.^[4]^ However, the esophageal surgery is still associated with high complication rates comparing with other gastrointestinal malignancies.^[17]^ Following esophagectomy, uncomplicated anastomosis is critically important for an uneventful postoperative course. Actually, some controversies still exist in the surgical process which includes operative approach, radicality of lymphadenectomy, and methods of reconstruction. There are several options for the reconstruction after esophagectomy such as specifics of conduit construction (whole stomach or gastric tube), site of anastomosis (intrathoracic or cervical) and anastomotic method (hand sewn or stapled).^[18–20]^ It is therefore not surprising that the merits and negative aspects of hand-sewn vs stapled esophagogastric anastomosis remain incompletely defined.

The hand sewn esophagogastric anastomosis can be constructed in an end-to-end or end to side fashion using single or double or even 3 layered suture. The mechanical esophagogastric anastomosis can be performed by using circular or linear staples. The circular stapled anastomosis was released in 1979 by Ravitch et al^[21]^ and has become increasingly popular since the 1990s. The linear stapled anastomosis was first described by Collard et al in 1998^[22]^ and was later modified by Orringer et al in 2000.^[23]^ The hand-sewn anastomosis depends more on the surgeon ability and is economic than stapled anastomosis. The stapled anastomosis is usually considered to allow for the uniformity of procedures and a short surgery time. To some extent, the stapled anastomosis could decrease the incidence of anastomotic leakages and postoperative mortality though the anastomotic stricture becomes more common. Many RCTs have been conducted to compare the hand-sewn and mechanical anastomosis methods. But the results still remain controversy. Luechakiettisak and Kasetsunthorn analysis 117 EC patients and conclude that hand-sewn and the staple methods were both safe. The stapled method had a higher incidence of anastomotic stricture whereas it brought less operative time and less blood loss.^[9]^ Harustiak et al retrospective reviewed 451 EC patients who received Ivor Lewis oesophagectomy and revealed that side-to-side linear-stapled technique is more preferred than hand-sewn technique method that could decrease the rate of overall anastomotic leak and anastomotic stricture.^[24]^ However, Rostas et al prospectively reviewed 142 EC patients and found the method of anastomotic construction had no bearing on the rate of complications. At the same time, the rate of symptomatic dysphagia which needed for dilations was equal between different groups.^[25]^

Several limitations have to be addressed in our review. First, we will only include studies published in English which may cause the publication bias in our study. Second, some confounding factors may exist in the review and meta-analysis such as different resources of patients and pathological types, different tumor stages and treatment strategies. These factors will increase the heterogeneity in our study. We will conduct Begg funnel plot and Egger test to evaluate the publication bias and undertake the subgroup analysis to explain the heterogeneity. But these limitations may hinder the interpretation of the results in the clinical work. Further meta-analyses including additional studies and increased sample sizes are needed to correct for publication bias and heterogeneity and improve the accuracy.

## Author contributions

**Conceptualization:** Xiangwei Zhang, Xianbiao Xue.

**Data curation:** Yang Wang, Xiangwei Zhang, Guoyuan Ma, Zhaoyang Wang, Xianbiao Xue.

**Funding acquisition:** Xiangwei Zhang, Lin Zhang.

**Investigation:** Yuanzhu Jiang, Zhaoyang Wang, Xianbiao Xue.

**Methodology:** Yang Wang, Yuanzhu Jiang, Xianbiao Xue.

**Software:** Yang Wang, Yuanzhu Jiang, Guoyuan Ma, Zhaoyang Wang, Xianbiao Xue, Shaowei Sang.

**Validation:** Guoyuan Ma, Shaowei Sang, Lin Zhang.

**Visualization:** Lin Zhang.

**Writing – original draft:** Yang Wang, Xiangwei Zhang.

**Writing – review & editing:** Shaowei Sang, Lin Zhang.
